# The Multiple Faces of Integrin–ECM Interactions in Inflammatory Bowel Disease

**DOI:** 10.3390/ijms221910439

**Published:** 2021-09-28

**Authors:** Valentina Garlatti, Sara Lovisa, Silvio Danese, Stefania Vetrano

**Affiliations:** 1 IRCCS Humanitas Research Hospital, Rozzano, 20089 Milan, Italy; valentina.garlatti@humanitasresearch.it (V.G.); sara.lovisa@humanitasresearch.it (S.L.); silvio.danese@hunimed.eu (S.D.); 2 Department of Pharmaceutical Sciences, University of Piemonte Orientale ‘A. Avogadro’, 28100 Novara, Italy; 3 Department of Biomedical Sciences, Humanitas University, Pieve Emanuele, 20072 Milan, Italy

**Keywords:** integrin, inflammatory bowel disease, IBD, intestinal fibrosis, inflammation

## Abstract

Inflammatory Bowel Disease (IBD) comprises a series of chronic and relapsing intestinal diseases, with Crohn’s disease and ulcerative colitis being the most common. The abundant and uncontrolled deposition of extracellular matrix, namely fibrosis, is one of the major hallmarks of IBD and is responsible for the progressive narrowing and closure of the intestine, defined as stenosis. Although fibrosis is usually considered the product of chronic inflammation, the substantial failure of anti-inflammatory therapies to target and reduce fibrosis in IBD suggests that fibrosis might be sustained in an inflammation-independent manner. Pharmacological therapies targeting integrins have recently shown great promise in the treatment of IBD. The efficacy of these therapies mainly relies on their capacity to target the integrin-mediated recruitment and functionality of the immune cells at the damage site. However, by nature, integrins also act as mechanosensitive molecules involved in the intracellular transduction of signals and modifications originating from the extracellular matrix. Therefore, understanding integrin signaling in the context of IBD may offer important insights into mechanisms of matrix remodeling, which are uncoupled from inflammation and could underlie the onset and persistency of intestinal fibrosis. In this review, we present the currently available knowledge on the role of integrins in the etiopathogenesis of IBD, highlighting their role in the context of immune-dependent and independent mechanisms.

## 1. Introduction 

Inflammatory Bowel Disease (IBD) defines a group of chronic, relapsing, and remitting enteropathies, including Crohn’s disease (CD) and Ulcerative colitis (UC) [1]. The leading causes of IBD onset and progression are poorly understood. However, it is well known that genetic susceptibility, alteration in the gut microbiota compositions, and environmental factors are strongly involved [2]. This multifactorial scenario sustains a chronic and aberrant immune response, triggering extensive intestinal tissue damage at the inflammatory site [3,4,5]. While in physiological conditions the tissue damage response leads to the correct restoration of the tissue homeostasis [6,7], the persistency of the inflammatory insult during IBDs triggers an unbalanced wound healing response resulting in chronic tissue damage and fibrosis, ultimately leading to organ dysfunction [8]. Intestinal fibrosis is a common complication in IBD and can occur in both UC and CD, although it is more prevalent in CD [9]. Tissue fibrosis is responsible for the onset of common IBD complications, such as strictures formation, also termed stenosis, which narrows the intestinal lumen and impairs bowel functionality [10,11,12].

Current anti-inflammatory therapies for IBD patients do not prevent or solve stenotic formations, especially in the latest stages of the pathology progression. These anti-inflammatory biological molecules can modulate inflammation but are ineffective in treating fibrosis onset and progression [13,14]. Therefore, despite the availability of these novel biological treatments, the incidence of strictures formation has not changed and requires the development of different therapeutic strategies [15]. This evidence suggests that IBD and its related complications, among which fibrosis is the most debilitating, are not only governed by inflammation but might be sustained and perpetuated by additional inflammation-independent mechanisms which need to be understood in depth and taken into consideration to develop new therapeutic strategies able to target these stenotic complications [16].

Molecular players that have recently become prominent in IBD research and clinic are represented by members of the integrin family [17,18]. Many studies have reported the central role of integrins in the recruitment of immune cells at the damaged tissue area and in supporting the progression of inflammatory mechanisms [17,19]. In addition, integrins exert fundamental roles in the cell interaction with the ECM, in both sensing and transducing the modifications of the extracellular milieu, and acting as mechanosensitive molecules. For this dual role in participating in both inflammation-dependent and -independent mechanisms, integrins have been investigated in the context of the intestinal disease and other organ districts [19].

In this review article, we aim to summarize the involvement of integrins in IBD-associated intestinal fibrosis development, taking into consideration their functional role in both immune cells and other cell types, primarily fibroblasts, highly implicated in the development of fibrotic complications.

## 2. Integrins: A Brief Overview

**Figure 1:**
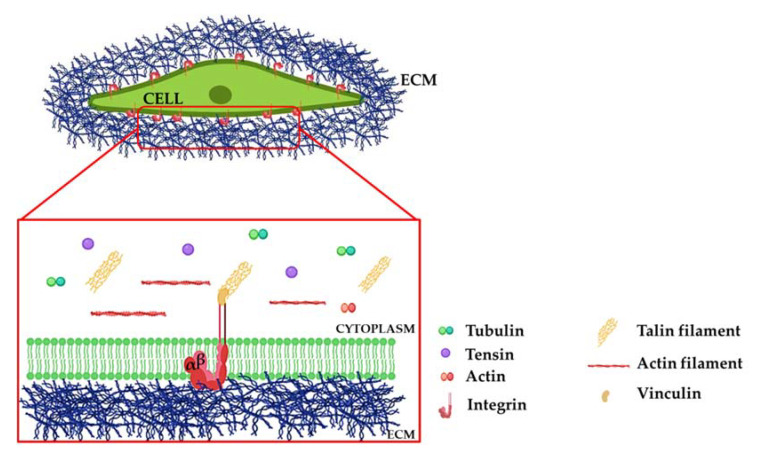
**Force transmission through integrins.** Schematic representation of a mesenchymal cell expressing integrins and ECM molecules. Integrins transduce messages from the extracellular matrix (ECM) to the actin cytoskeleton by providing a link across the plasma membrane. Any force applied to the ECM leads to ECM molecule rearrangement and is transmitted as a mechanical signal to ligand-bound integrins. The zoom represents the positioning of integrins with respect to ECM components. Integrins do not directly communicate with the cytoskeleton. However, they are composed of a cytoplasmic domain called the tail, connected to actin-binding adaptor proteins such as talin, tensin, or actin, which transmit forces from the integrins to the cytoskeleton.

Integrins are heterodimeric transmembrane receptors composed of α and β subunits acting as a bridge between the ECM and the cellular cytoskeleton [20,21]. Integrins are about 90–120 kDa molecules characterized by a multi-domain molecular structure, with a large extracellular domain linked through a transmembrane portion to a cytosolic tail directly interacting with the cytoskeleton [18,21,22]. The integrin family consists of 18 alpha and 8 beta subunits that can combine to form 24 different homo- or heterodimeric receptors, which bind specific ligands [23]. β-subunits are characterized by a cytoplasmic binding site involved in recognition of cytoskeletal cell components and transduction of downstream signalling. In contrast, the α-domains are engaged in establishing ligand type specificity with the outer ECM components [24]. In recent years, it has become clear that integrins have profound effects on the development of various diseases such as cancer and fibrotic diseases [19,25]. Moreover, many of the key cell–cell and cell-matrix interactions that regulate these pathologies are mediated by members of the integrin family. Collagens, laminins, and RGD-domains represent the preferential extracellular binding partners of integrins. In addition, through their cytosolic domain, integrins can cooperate with various intracellular proteins such as talin, vinculin, and actin (Figure 1) [26].

### Molecular Mechanisms of Integrin Signaling

**Figure 2:**
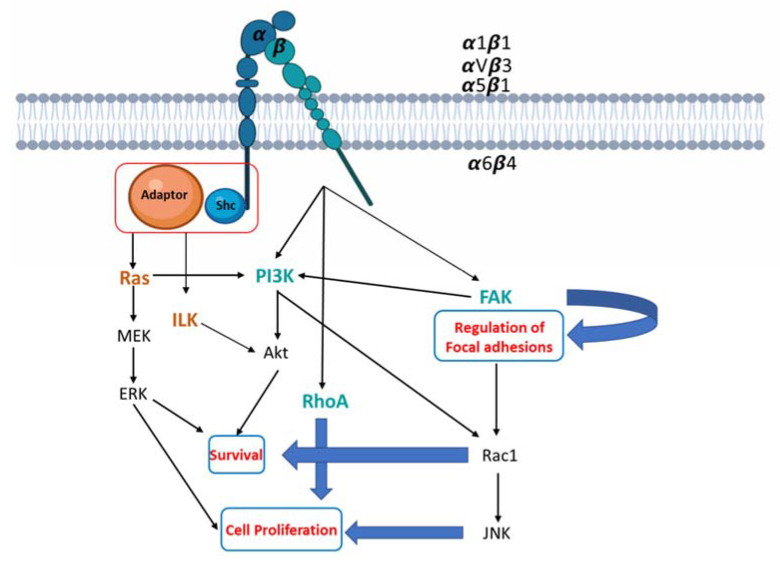
**Integrin-mediated signaling pathways leading to cell proliferation and survival.** Schematic representation of the multiple signaling pathways activated by integrins and affecting cell proliferation and survival. Integrins regulate cell survival via Ras/MEK/ERK, ILK/Akt (both requiring the Shc subunit and an adaptor protein), and PI3K (through Akt or Rac1) signaling pathways. In addition, proliferation is mediated by Ras/MEK/ERK, RhoA, PI3K, and FAK, the latter also being involved in the regulation of focal adhesions.

Integrin affinity for ECM molecules stabilizes the connection between the cell cytoskeleton and the ECM, activating intracellular signaling pathways involved in regulating biological functions such as proliferation, apoptosis, and differentiation [27]. This interaction starts with the translocation of integrins from the cytosol to the plasma membrane and the subsequent creation of integrin clusters sensing the extracellular microenvironment and transducing external signals [28,29]. This activation, in turn, triggers the activation of the downstream molecules focal adhesion kinase (FAK), proto-oncogene non-receptor tyrosine kinase (SRC), serine-threonine protein kinase (AKT), and small GTPases belonging to the Rho family [29] (Figure 2).

Signaling pathways triggered by integrin activation are often the same activated by soluble molecular factors. For example, epidermal growth factor (EGF), platelet-derived growth factor (PDGF), and lipoprotein A (LPA) require cell adhesion to ECM, a function that is exerted by integrins. This explains why integrins and soluble molecular factors share common signaling axes [30].

In addition, integrins play a crucial role in maintaining a balance between apoptosis and cell proliferation in normal cells. In the first case, the function is based on the activation of the phosphatidylinositol 3-kinase (PI3K) signaling pathway, and in the second is based on the extracellular signal-regulated kinase (ERK), cyclin D1 cascade, and cyclin E-cyclin dependent kinase (CDK) 2 complex in early G1 phase. Furthermore, the effect of the cyclin E-CDK2 complex seems to be mediated by the downregulation of the CDK inhibitors p21 and p27. These findings suggest the involvement of integrins in regulating cell cycle progression [31,32].

Another fundamental function regulated by integrins is cell motility. Integrins influence the phosphorylation status of the cell motility machinery, enhancing the activation of the myosin light chain kinases (MLCK) and the phosphorylation of the myosin light chain (MLC) molecule [33]. It has been studied that ERK can also phosphorylate MLCK, inducing MLC activation in a calmodulin-dependent manner [34,35]. In addition, survival signaling pathways are associated with both growth factor receptors and cell adhesion molecules, and are relevant in protecting cells from pathological and homeostatic cell death. For example, molecular growth factors such as EGF, PDGF, and insulin can induce survival of serum-starved epithelial cells [36,37,38]. At the same time, binding of integrins such as αvβ3 [39], α5β1, and α6β1 [40,41,42] to the appropriate extracellular matrix protein can inhibit cell death.

## 3. The Role of Integrins in Inflammation-Dependent Mechanisms in IBD

Homing of immune cells to the injured tissue is mediated by chemo-attractive mechanisms and facilitated by interactions between integrins expressed by leucocytes and adhesion molecules presented on the endothelial cell surface. Various preclinical studies have elucidated the critical role of leukocyte-expressed integrins in different inflammatory diseases, demonstrating that their deficiencies may significantly moderate both the pathogenesis and the development of IBDs [43].

Integrin heterodimers mainly involved in mediating the homing of lymphocytes to the gut mucosa are α4β1, α4β7, and αEβ7. α4β1 and α4β7 integrins are highly expressed on CD4^+^ memory T cells [44,45], and they interact, respectively, with the vascular cell adhesion molecule 1 (VCAM-1) expressed on the endothelium [46], and the mucosal addressin cell adhesion molecule 1 (MAdCAM-1) expressed in the gut-associated lymphoid tissues (GALT) [47]. The fundamental function of the α4 integrins in intestinal inflammation and immune cell recruitment was demonstrated early on by immunoblockade of α4 integrin in a cotton-top tamarin model of colitis [48,49]. Adoptive transfer of α4 null T cells in immunodeficient mice significantly attenuated chronic colitis and confirmed the defective homing of T cells to the inflamed intestinal mucosa [50].

MAdCAM-1 expression is largely restricted to the intestinal tissue, therefore its interaction with α4β7 integrin is responsible for most of the T cells homing in the gut. Both MAdCAM-1 and VCAM-1 were reported to be increased in inflamed intestinal biopsies from CD and UC patients [51,52], and this upregulation enhances the α4β1- and α4β7-mediated recruitment of inflammatory lymphocytes. MAdCAM-1 expression has also been reported on venules of lymphoid aggregates in CD patients [51]. Additionally, α4β7 expression facilitates the gut entry of regulatory T cells, while blocking α4β7 reduced the intestinal homing of both effector and regulatory T cells [53].

Parallel to the recruiting and homing functions of α4β1 and α4β7 heterodimers, the αEβ7 integrin instead exerts its major role in retaining T cells and dendritic cells in the intestinal mucosa. The expression of αEβ7 on the surface of T lymphocytes, induced by the local TGFβ [54,55], allows these immune cells to be retained in the intestinal epithelial layer thanks to the engagement with its ligand E-cadherin [56,57,58]. The pro-colitogenic role of the αEβ7-expressing lymphocytes is well documented. αEβ7 was expressed by a subset of pro-inflammatory colonic CD4^+^ T cells, which display Th1 and Th17 inflammatory profile and reduced expression of regulatory T cell-associated genes such as FOXP3, IL-10, CTL-4, and ICOS [52]. Furthermore, αE expression on a subset of resident memory CD4^+^CD69^+^ T cells, which accumulate in the mucosa of IBD patients, is predictive for the development of flares [59]. αEβ7 was also shown in CD to be expressed on an expanded subset of CD4^+^ T cells expressing the NKG2D receptor and displaying inflammatory and cytotoxic properties [60]. Elevated expression of αEβ7 was noted on Th9 CD4^+^ and CD8^+^ cells, in comparison to α4β7 being expressed by the Th2 and Th17 T cells [55]. In addition to CD4^+^ and CD8^+^ cells, αEβ7 was found upregulated on a subset of γδ-T lymphocytes found in the mesenteric lymph node and the intestinal tissue in a mouse model of T cell-mediated colitis and the SAMP spontaneous chronic ileitis [61]. In addition to being physically retained in the epithelium, a direct cytotoxic activity of the αEβ7-expressing T lymphocytes on the intestinal epithelial cells has been reported [52,62].

In line with the functional role of αEβ7 highlighted by all these studies, administration of a monoclonal antibody against αEβ7 (Table 1) was able to reduce the accumulation of CD4^+^ T cells in the lamina propria and their IFNγ production, thus ameliorating immunization-induced colitis in the IL2 knockout mouse model [63]. Moreover, etrolizumab-mediated blockade of αEβ7 was more effective than vedolizumab-mediated targeting of α4β7 in reducing the colonic numbers of CD8^+^ and Th9 cells [55].

Integrin αE is also expressed in the dendritic cells (DCs) in the subepithelial lamina propria and the mesenteric lymphnodes [64,65]. In physiological conditions, these αE^+^ DCs exert a tolerogenic function in the regulation of T cell homing [66,67]. The frequency and functionality of αE^+^ DCs were reported to be altered in the inflamed intestinal mucosa [68]. Specifically, the function of αE^+^ DCs was shown to be altered in the colon of UC patients. These DCs displayed an impaired capacity to generate regulatory T cells and induced a Th1/Th2/Th17 phenotype in CD4^+^ effector T cells [69].

Overall, these studies highlighted the integrin-mediated infiltration and retention of immune cells, as well as their switch to a pro-inflammatory phenotype, as fundamental mechanisms underlying IBD pathogenesis.

## 4. Anti-Integrin Therapies for IBD Based on Immune-Dependent Functions

The finding that integrin-mediated leucocytes trafficking plays a crucial role in IBD and could therefore represent a valuable therapeutic strategy has led to the development of monoclonal antibodies against leukocyte-expressed integrins. In contrast to the traditional immune-suppressive therapies, certainly able to modulate the inflammatory response but also generating harmful immunosuppression, anti-integrin therapies offered the opportunity to develop a more targeted approach, thus overcoming potential systemic side effects. In line with the knowledge acquired through in vitro and in vivo studies, the most diffused and effective anti-integrin treatments currently available are directed against integrins α4β1, α4β7, and αEβ7 (Table 1).

Natalizumab is a humanized monoclonal IgG4 antibody against the α4-integrin, acting on both α4β1 and α4β7 heterodimers. It inhibits the interaction between α4β7 and endothelial MAdCAM-1, blocking lymphocytes recruitment in the gastrointestinal lymphoid tissue [70]. Phase II and III studies show that natalizumab is effective as an induction and maintenance therapy in CD patients [71,72] and it has usually been used in patients not responding to other previously administered treatments [73]. However, its use has been limited due to the reported risk of Progressive Multifocal Leukoencephalopathy (PML), a rare but potentially fatal demyelinating brain disorder caused by polyomavirus JC which commonly occurs in HIV positive people, but is also reported in patients receiving long-term immunosuppression [74,75,76]. Natalizumab is thought to increase PML risk by preventing lymphocytes from adhering to the endothelium of the blood–brain barrier, therefore reducing their migration and suppressing T cell-mediated immune response in the brain [77].

Vedolizumab is a humanized monoclonal immunoglobulin G1 against α_4_β_7_ integrin. Its role in modulating lymphocytes trafficking is exerted in the gut but not in the brain, avoiding the predisposition to PML [78]. Benefits in patients with IBD have been elucidated in phase II clinical trials in patients with moderately to severely active Crohn’s disease [79].

Etrolizumab is another humanized monoclonal antibody selectively binding the β7 subunit of both α4β7 and αEβ7 heterodimers, antagonizing both α4β7-MAdCAM mediated lymphocyte recruitment and the αEβ7-E-cadherin interaction [80], which mediates T cells adhesion to epithelial cells [81]. Results of phase II clinical trials indicated that etrolizumab as induction therapy leads to a significant higher proportion of clinical remission compared to placebo, in patients with moderate-to-severe UC [82].

AJM300 is a small molecule orally administered and able to antagonize α_4_ integrins. Promising outcomes have been illustrated from several preclinical studies of experimental IBD animal models such as the DSS- and TNBS-induced. In 2009, Takazoe et al. reported the results obtained during a randomized study involving Japanese active CD-affected patients treated with AJM300 compound or placebo [83]. The clinical trial adopted AJM at three different doses (40 mg three times a day (TID), 120 mg TID, or 240 mg TID), or placebo, orally for eight weeks. AJM300 was safe and well-tolerated. However, the study was not pursued as no significant difference between the AJM300 treatment groups and the placebo group was reported. AJM300 evaluation efficacy, therefore, requires further investigation [83].

Abrilumab is an anti-α4β7 monoclonal antibody acting on immune cell trafficking, used in phase IIb clinical trials. Abrilumab can inhibit leukocytes recruitment and extravasation from the blood vessels and a specific sub-population of CD4^+^T cells (CD4^+^T-CD45RA^−^). Nowadays, Abrilumab has been tested in UC patients, specifically in refractory patients with mild to severe UC. In fact, in 2019, Sandborn et al. tested the anti-α4β7 integrin monoclonal antibody at different doses (7, 21, 70, and 210 mg) in UC patients through subcutaneous administration [84]. Significant clinical responsiveness and mucosal healing were observed at week 8 of treatment, in the regimen with repeated 70 mg dosages and the single 210 mg subcutaneous administration, suggesting its promising potential as a new treatment for IBD patients [84].

## 5. Integrins in IBD-Associated Fibrosis: A Key Player in Its Onset, Independent of Inflammation

### 5.1. Fibrosis and Tissue Stiffness

Anti-inflammatory therapies for IBD are ineffective in treating pathological consequences of chronic inflammation such as fibrosis development. Despite stenotic complications, usually following the IBD pathology’s relapsing and remitting inflammatory fluctuations, it seems that intestinal fibrosis can partly occur independently from inflammation at the later stages of the disease, and that biologicals display some effects in inflammatory, but not fibrotic, strictures [13,14,85]. This can be explained by an auto-propagation of the fibrotic mechanisms supported by the persistent interaction between cellular compartments and the ECM matrix. The dysregulated release of ECM molecules supports the increase in matrix stiffness, a prominent feature of CD-associated intestinal fibrosis [86]. During homeostatic conditions, matrix stiffness degree is tightly regulated by the balance between deposition of ECM and tissue remodeling operated by enzymes such as metalloproteinases (MMPs) [87]. However, matrix deposition and turnover can be highly subverted during injury and CD-associated tissue damage, leading to a pathological scenario [88]. Matrix stiffness has been reported to be one of the mechanical forces sensed by cells able to modulate many biological functions, such as cell growth, survival, and motility. It is documented that an increment of matrix stiffness can modulate cell proliferation and differentiation [89]. Cells, especially related to the epithelial and mesenchymal compartment, can sense mechanical stiffness derived from the outer ECM and the adjacent cells. The transduction of extracellular mechanical stimuli and conversion of those signals into downstream signaling pathways is referred to as mechanotransduction and has been deeply studied in the recent decade in the field of fibrosis [90]. The mechano-signaling is a finely regulated process that relies on various proteins such as integrins and focal adhesion molecules, intracellular and cytoskeletal proteins, and nuclear responders [91]. Genetic ablation of α6 integrin in collagen-expressing mesenchymal cells or pharmacological blockade of matrix stiffness-regulated α6-expression was shown to protect mice against experimental lung fibrosis [88]. These findings suggest that integrins may be considered matrix stiffness-regulated mechanosensitive molecules that orchestrate the invasive fibroblast phenotype and mediate experimental lung fibrosis. Therefore, an alteration of matrix stiffness degree is sensed by integrins, leading to aberrant activation of cellular biological functions, eventually leading to tissue dysfunction, such as intestinal fibrosis. Intestinal fibrosis is characterized by increased tissue stiffness, distinguished from a healthy bowel by mechanical measurement and ultrasound elasticity imaging [92]. Ex vivo colonic tissue from normal, strictured, and the unaffected surgical margin was used to determine the stiffness of fibrotic compared to the normal colon for the development of an in vitro cell culture model of tissue stiffness [93]. This study showed that CD-associated strictured colon is nearly 6-fold stiffer than the normal bowel or the unaffected margin. Moreover, expression of genes involved in fibrogenic matrix remodeling, barrier function, and inflammatory was significantly increased in the CD strictures, therefore indicating concurrent fibrosis and inflammation. It has been demonstrated that increased matrix stiffness associated with the fibrostenotic disease alters colonic myofibroblast activation, producing a fibrogenic phenotype and auto-propagating fibrosis [94]. Indeed, activated colonic myofibroblasts function as key mediators of fibrosis and tissue stiffness by two mechanisms: (1) excessive extracellular collagen production which stiffens the ECM and (2) active contraction of the ECM, which exerts additional mechanical force on the matrix [95].

### 5.2. The Role of ECM in Intestinal Fibrosis: Not Only Support but Also a Reservoir of Molecular Mediators 

The ECM is a three-dimensional molecular structure that provides an architecture to the organ tissue. Its molecular composition serves as a ligand for cell membrane receptors, such as integrins and various adhesion molecules [22]. The relevance of ECM has been demonstrated through many in vivo loss of function studies, in which genes encoding for fundamental ECM proteins have been silenced, leading to the onset of several pathologies characterized by mice mortality at early embryonic stages [96,97]. The ECM is composed of over 300 proteins, including laminins, fibronectin, and collagens. These molecules are the most representative, and their crucial role has been demonstrated in various diseases, supporting the idea that ECM balance is essential in all organs [98]. The interstitial ECM is mainly produced by cells belonging to the mesenchymal cell compartment. Differently, basement membrane-ECM plays a role in maintaining the epithelial cell layer and spatially separates it from the underlying intestinal *lamina propria*. The interstitial ECM is the most fundamental in fibrosis development. It contains molecular fibrotic mediators, such as epidermal growth factor (EGF), fibroblast growth factor (FGF), and TGFβ in a latent state. Among those, the most documented is the activation of TGFβ, the principal molecular factor involved in fibrosis development. Integrins and the downstream focal adhesion (FAK) complex proteins, known as mechano-sensors and mechanotransducers [23], sense and transduce external mechanical stimuli into biochemical signals [99]. The downstream signalling pathways induced by integrin-FAK complexes in response to matrix stiffness is bidirectional, such that there is a close relationship between the matrix, which exerts force on the cell via integrins, and the cellular cytoskeleton, which in turn resists this force [100].

### 5.3. Mechanisms of Integrin-Induced Release of Latent TGFβ

Integrins are involved in the direct activation of soluble molecular factors, which in homeostatic conditions are imprisoned in a latent form by the ECM network. In intestinal fibrosis, integrins have been involved in activating latent TGFβ [25,101,102], which is highly secreted by CD-associated stricture myofibroblasts [103]. TGFβ molecule is released in the extracellular microenvironment and conserved in a latent state at high concentration and directly cross-linked to the ECM. TGFβ activation is carried out by the interaction of integrins to a linear arginine-glycine-aspartic acid (RGD) motif present in an N-terminal fragment of TGFβ called a latency-associated peptide (LAP) [104].

Integrin-mediated TGFβ activation can be carried out in a protease-dependent or protease-independent manner. Integrin αvβ8 has been shown to bind to the RGD peptide in LAP and subsequently recruits MMP14, which then releases TGFβ by proteolytic cleavage. Similarly, integrin αvβ3 can act as a docking site for MMP2 and MMP9 [105].

**Figure 3:**
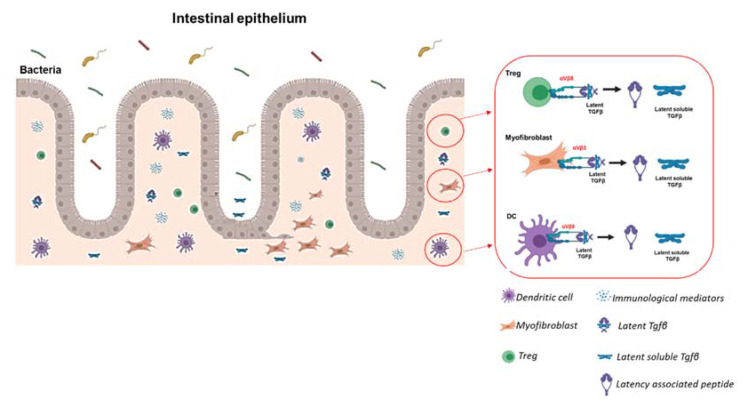
**Latent and active TGFβ in IBD**. TGF-β production as a soluble ligand is sustained by immune cells (mainly dendritic cells (DCs) and regulatory T cells (Tregs)) and other cell compartments such as epithelial cells and fibroblasts. TGF-β is usually imprisoned in a latent form in the ECM by association with the latency-associated peptide (LAP). Following ECM remodeling, latent TGF-β is converted into the functionally active form by the action of αvβ3, αvβ6, and αvβ8 integrins. As a result, released TGF-β can bind to its receptors, usually residing on immune cells (T-cells, B-cells, DCs, and macrophages), epithelial cells, and fibroblasts.

Non-proteolytic activation instead occurs through cell traction forces due to increased matrix stiffness as it happens during fibrosis. The increased matrix stiffness provokes a tension of the cellular cytoskeleton, which modifies the interaction between integrin and the latent-TGFβ. This connection can cleave LAP and release the mature growth factor in the extracellular microenvironment [104]. Furthermore, these forces stimulate integrins to induce conformational changes of the TGFβ–LAP–LTBP complex, by which active TGFβ is released and able to interact with its receptors directly [106]. Interestingly, a recent study highlighted that activation of TGFβ by αvβ8 can occur independently from cytoskeletal forces and does not require the release from the latent peptide [107] (Figure 3).

Several integrins have been investigated in the pathogenesis of fibrosis in many organs, and their role in activating latent TGFβ has been well elucidated [25]. αv and α5 are the major group of integrins detected in intestinal fibrosis, especially for their involvement in the activation of TGFβ. αvβ3, αvβ5, and αvβ6, and their role in activating TGFβ, have been very well demonstrated in many in vitro studies [106]. In in vivo studies, the upregulation of αvβ6 in the epidermis has provoked elevated TGFβ1 activation leading to chronic ulcers formations and severe fibrosis [108].

In support of this evidence, in vivo studies demonstrated that αvβ6 and αvβ8 ablation recapitulates TGFβ knockout mouse phenotype and mutations in the RGD peptide share similar alterations observed in the TGFβ knockout mouse model [109].

Latent TGFβ1 was also reported to be activated by the αvβ3 RGD domain expressed in human and rat intestinal smooth muscles [110], and that an αvβ3 RGD inhibitor could be a novel treatment to diminish excess TGF-β1 activation, collagen I production, and development of fibrosis in CD [111]. In addition, an interaction between TGFβ, Smad3, αvβ6, and mTOR was observed in the development of intestinal fibrosis [112]. Integrins can also potentiate the action of the TGFβ pathway, supporting the over-expression of its downstream related genes. For example, the over-expression of α3β1 was shown to enhance MMP9 expression levels in keratinocytes [113], and the up-regulation of integrin β1 was demonstrated to induce p38/MAPK signaling during the EMT process [114]. Moreover, SMAD2 and SMAD3 cascade were found to be reduced as a consequence of αV blocking [115].

## 6. Functional Contribution of Integrins Expressed by Fibrosis-Associated Fibroblasts

Fibroblasts are active and fundamental players in the wound healing response and the progression to fibrosis [116,117]. By the fibroblast-to-myofibroblasts conversion process, these cells become activated and enabled to produce and deposit large amounts of ECM, which progressively accumulates during the fibrotic process. The fibrotic matrix itself, in turn, participates in the fibroblasts’ conversion through mechanotransduction processes [118].

A functional role for the fibroblast-expressed integrins has been demonstrated in multiple fibrotic disease models. To date, most of the work has been focused on the αV subunit [119]. Fibroblasts can express four types of αv heterodimers: αvβ1, αvβ3, αvβ5, and αvβ8. Conditional deletion of the *Itgav* gene (encoding integrin αv) in Pdgfrβ^+^ myofibroblasts was shown to protect mice from tetrachloride-induced hepatic fibrosis [120]. In vitro experiments using genetic or pharmacological inhibition of αv integrin confirmed the reduction in the profibrotic phenotype (collagen I and III, and αSMA expression) in these fibroblasts. Additionally, genetic deletion of αv integrin exerted a protective role also in bleomycin-induced pulmonary fibrosis and Unilateral Ureteral Obstruction (UUO)-induced renal fibrosis [115]. The high expression of αvβ1 mediates fibroblasts’ adhesion to LAP and activation of latent TGFβ [25,101,121]. The relevant heterodimer involved in this context was speculated to be αvβ1 as the global or conditional knockout of all the other possible binding partners (β3, β5, β6, and β8) failed to protect against hepatic fibrosis. The pre-clinical use of the αv small molecule inhibitor CWHM12 demonstrated to be effective in attenuating liver and lung fibrosis, even when fibrosis was already established [115], and the subsequent design of an αvβ1 small molecule inhibitor further proved the efficacy of targeting this integrin complex [101].

A functional role for fibroblast-expressed integrin αvβ8 was shown in the lung disease where conditional deletion or neutralization of integrin β8 in lung fibroblasts inhibits airway inflammation and fibrosis [44,122]. Furthermore, the αvβ8-mediated activation of TGFβ by fibroblasts was shown to be crucial for directing dendritic cell trafficking [44]. The fact that αvβ8 functionality in fibrosis-associated fibroblasts may be organ-dependent is indicated by the fact that conditional knockout of β8 in the hepatic fibroblast did not exert any protection from hepatic fibrosis.

αvβ3 expression in intestinal smooth muscle cells leads to activation of latent TGFβ and subsequent collagen production and fibrosis in the *muscolaris mucosa* in the TNBS-induced experimental model of intestinal fibrosis [123]. The RGD-containing αvβ3 integrin inhibitor Cilengitide administration decreased TGFβ1 activation, collagen production, and diminished TNBS-induced fibrosis.

Compared to the other αv-containing heterodimers, the αvβ6 integrin is upregulated exclusively in epithelial lineages during an injury in multiple models. αvβ6 expression has been reported in the renal epithelium [124], cholangiocyte and hepatic progenitors [125,126], pneumocytes, and alveolar epithelium [127,128]. In all these studies, genetic and pharmacological blocking of αvβ6 proved effective in reducing lung, kidney, and biliary fibrosis. All these models demonstrated that the activation of latent TGFβ mediates the mechanism of αvβ6-induced fibrogenesis. In addition, αvβ6 was shown to be up-regulated in the submucosa and serosa of TNBS treated mice [112]; however, no detailed investigation on its precise expression and functionality in the intestinal epithelium has been conducted.

## 7. Future Perspectives

Despite significant advancement in the treatment of IBD obtained with immunomodulators, there is still a substantial portion of IBD patients who are refractory or acquire resistance to these therapies. Targeted anti-integrin therapies represent a promising therapeutic option for managing the disease in this fraction of patients [17]. The therapeutic strategy underlying these anti-integrins is represented by blocking the functionality of integrins expressed by immune cells and mediating their homing and retention to the damaged intestine [17].

It has become increasingly appreciated that inflammation is not the only component of IBD disease. The majority of complications, such as strictures and stenosis, arise due to fibrotic processes, particularly in Crohn’s patients [129,130]. The fact that anti-inflammatory therapies are substantially ineffective towards these fibrotic complications suggests that, although it might indeed arise as a consequence of inflammation, once established, the fibrotic response could progress and self-perpetuate independently from inflammation, as also suggested by mounting experimental evidence [131,132]. Fibroblasts represent the major cell type involved in the establishment and evolution of fibrosis. As reviewed above, various types of integrin heterodimers are expressed by fibrosis-associated fibroblasts, and their functional involvement has been demonstrated in multiple fibrotic disease models, including liver, kidney, and lung [101,115]. On the contrary, the phenotypic and functional characterization of integrin expression in the fibrotic fibroblasts associated with intestinal fibrosis is very limited and would certainly deserve further investigation.

The vast majority of the studies utilizing genetic or pharmacological inhibition of fibroblast-expressed integrins have identified the activation of the latent TGFβ peptide as the major mechanism responsible for the induction of the integrin-mediated pro-fibrotic phenotype [101,133]. In this regard, numerous open questions remain to be answered concerning the intracellular signaling pathways engaged as a consequence of the activation of the integrin signaling in fibroblasts. Moreover, considering that integrins function as mechano-transducers translating stimuli from the external ECM into intracellular changes, understanding the functional role of fibroblast-expressed integrins from a mechanotransduction perspective may shed some light on the complex reciprocal interplay between intestinal matrix remodeling and fibroblasts functionality. This would potentially provide insights into the mechanisms of fibrosis propagated by tissue remodeling events and uncoupled from inflammatory processes and, in turn, explain why blocking intestinal inflammation alone is ineffective in limiting fibrotic processes.

## 8. Closing Remarks

Considering that multiple intracellular pathways are activated by integrins, and the way that ECM changes modulate much fibroblast functionality, it is possible to predict that the activation of the latent TGFβ is not the only molecular pathway activated by fibroblast-expressed integrins. Extending our understanding of the complex interplay between inflammation and tissue remodeling in IBD to the multiple cell types potentially involved, and considering the fibrotic matrix as an active player rather than just a scaffold may provide novel mechanistic insights to further our comprehension of the disease and design novel therapies for the IBD-associated fibrotic complications.

## Figures and Tables

**Table 1:** Anti-integrin therapies in IBD.

Drug	Target	Clinical Trials	Indication in IBD
*NATALIZUMAB*	α4 integrin	ENCORE	Induction and maintenance in CD
*AJM300*	α4 integrin	Phase IIa	Induction in UC
*VEDOLIZUMAB*	α4β7 integrin	Gemini1, Gemini2, Gemini3	Induction and maintenance in UC (Gemini1) Induction and maintenance in CD (Gemini2,3)
*ETROLIZUMAB*	β7 integrin	Eucalyptus, Bergamot, Hickory	Induction in UC (Eucalyptus) Induction in CD (Bergamot) Induction in CD (Hickory)
*ABRILUMAB*	α4β7 integrin	Phase IIb	Induction in UC and CD

UC: Ulcerative Colitis; CD: Crohn’s Disease.

## Data Availability

Not applicable.
